# Efficacy and safety of Traditional Chinese Medicine injections for no-reflow or slow flow in patients with acute coronary syndrome after percutaneous coronary intervention: a systematic review and network meta-analysis

**DOI:** 10.3389/fcvm.2025.1619345

**Published:** 2026-01-07

**Authors:** Tongze Cai, Wanying Li, Wenhua Xu, Caiyue Lin, Liuguan Liang, Ding Wang, Jinghui Zheng

**Affiliations:** 1 Department of Oncology, Ruikang Hospital Affiliated to Guangxi University of Chinese Medicine, Nanning, China; 2 Graduate School, Guangxi University of Chinese Medicine, Nanning, China; 3 Department of Science and Technology, Ruikang Hospital Affiliated to Guangxi University of Chinese Medicine, Nanning, China; 4 Byherbs Orthopedics Clinic of Traditional Chinese Medicine, Nanning, China; 5 Office of Academic Affairs, Guangxi University of Chinese Medicine, Nanning, China

**Keywords:** percutaneous coronary intervention, no-reflow or slow blood flow, traditional Chinese medicine injection, network meta-analysis, ischemia-reperfusion injury

## Abstract

**Introduction:**

: There is a possibility of ischemia-reperfusion injury following percutaneous coronary intervention (PCI). Traditional Chinese medicine (TCM) treatment features multi-target and multi-link interventions, along with relatively good safety profiles, making it a promising option for alleviating ischemia-reperfusion injury following PCI.

**Methods:**

We searched the China National Knowledge Infrastructure (CNKI), VIP Journal Database, China Biomedical Literature Database, Wanfang Medical Database, PubMed, Web of Science, Embase, and Cochrane Library for randomized controlled trials published in Chinese and English regarding Chinese herbal injections for treating no-reflow or slow-reflow after PCI, from database inception to November 30, 2024.

**Results:**

A total of 14 interventions were evaluated, involving 5,535 patients. The interventions encompassed 14 types, including Shenfu Injection, Ginseng Injection, Shengmai Injection, Tanshinone IIA Sodium Sulfonate Injection, Danhong Injection, Danshen Injection, Shuxuetong Injection, Tanshinone Injection, Salvianolate Lyophilized Injection, Xuesaitong Injection, Salvia Ligustrazine Injection, Ixeris Sonchifolia Injection, Ginkgo Damo Injection, and standard treatment. The results indicated that the combination of salvianolate lyophilized injection with standard treatment might provide the best efficacy in improving no-reflow or slow blood flow after PCI. For enhancing Left Ventricular Ejection Fraction (LVEF) and reducing Left Ventricular End-Diastolic Dimension (LVEDD), the effect of Danshen Injection combined with standard Western medicine treatment was the most significant. Regarding reducing Left Ventricular End-Systolic Diameter (LVESD) and decreasing the incidence of Major Adverse Cardiovascular Events (MACE), Shenfu Injection showed possibly the most ideal effect. Meanwhile, Danhong Injection combined with standard treatment significantly improved the incidence of no-reflow compared to standard treatment, while also exhibiting positive effects on LVEF, reducing LVESD, and effectively decreasing the occurrence of MACE.

**Conclusion:**

Traditional Chinese Medicine (TCM) injections demonstrate significant efficacy and a favorable safety profile in treating no-reflow or slow blood flow after PCI in patients with acute coronary syndrome.

**Systematic Review Registration:**

https://www.crd.york.ac.uk/PROSPERO/view/CRD42024497449, PROSPERO CRD42024497449.

## Introduction

Acute Coronary Syndrome (ACS) is a group of clinical syndromes resulting from acute myocardial ischemia. Based on clinical presentation, electrocardiographic changes, and elevated cardiac troponin levels, ACS is categorized into unstable angina (UA), non-ST-segment elevation myocardial infarction (NSTEMI), and ST-segment elevation myocardial infarction (STEMI) (1, 2).

Percutaneous coronary intervention (PCI) is a crucial therapeutic approach for ACS that significantly improves myocardial blood flow and reduces mortality. However, complications such as the no-reflow phenomenon (NRP) and coronary slow-flow phenomenon (CSFP) can occur post-PCI, potentially enlarging the myocardial infarction area and worsening prognosis, thus increasing mortality and the risk of recurrent myocardial infarction (3). The incidence of NRP in selective PCI for Acute Myocardial Infarction (AMI) ranges from 0.6% to 6.0%, while it is higher (10.0% to 50.0%) in emergency settings (4). This underscores the substantial risk of NRP in this population and highlights the critical need for timely intervention to improve clinical outcomes. From the perspective of Traditional Chinese Medicine (TCM), no-reflow or slow flow after PCI in ACS patients falls under the categories of “chest bi” (chest obstruction) and “true heart pain”. The condition is primarily attributed to Qi Deficiency and Blood Stasis, often accompanied by Yang Collapse. The therapeutic principles are therefore to Supplement Qi, Warm Yang, Activate Blood Circulation, and Resolve Stasis. Modern pharmacological research has demonstrated that TCM injections developed under these principles exhibit multi-target effects, including anti-inflammatory, antiplatelet, antithrombotic, and vascular endothelial protective properties (5). These mechanisms align with the pathological processes of no-reflow, such as microvascular obstruction and ischemia-reperfusion injury, thereby providing a solid theoretical and mechanistic basis for the application of TCM injections in this context and for the subsequent network meta-analysis evaluating their comparative efficacy.

Current management strategies focus on dilating coronary and microvascular systems (6) and inhibiting platelet activation and aggregation to enhance myocardial perfusion and mitigate ischemia-reperfusion injury (7). Despite advancements in NRP management, the incidence of adverse cardiovascular events remains high. TCM offers a multi-target therapeutic approach with a favorable safety profile, making it a potential treatment strategy for no-reflow or slow flow post-PCI (5).

This study conducts a network meta-analysis to evaluate the efficacy of various TCM injections in reducing the incidence of no-reflow or slow flow after PCI for ACS and their role in minimizing adverse effects, thereby providing evidence to inform clinical practice and optimize treatment strategies.

## Materials and methods

### Retrieval strategy

According to the PRISMA guidelines (8), we conducted a systematic review, meta-analysis, and network meta-analysis of clinical trials related to treatment strategies for the incidence of no-reflow after PCI in patients with acute coronary syndrome. This evaluation protocol had been registered on the PROSPERO platform, with registration number CRD42024497449.

We searched for randomized controlled trials (RCTs) published in Chinese and English from database inception until November 30, 2024, we utilized the following databases: China National Knowledge Infrastructure (CNKI), VIP Journal Database, China Biomedical Literature Database, and Wanfang Medical Database, as well as English databases such as PubMed, Web of Science, Embase, and the Cochrane Library. The Chinese search terms for diseases included “No-Reflow Phenomenon”, “Slow-Flow Phenomenon”, “Acute Myocardial Infarction”, “Acute Coronary Syndrome”, and “Percutaneous Coronary Intervention”, The search terms for traditional Chinese medicine injections included “Chinese Medicine Injection”, “Traditional Chinese Medicine Injection”, and “Danhong Injection”, Research types were specified using terms such as “Randomized Controlled Trial”, “Randomized Controlled Study”, “Randomized Controlled Experiment”, and “Randomized Control”, The English search terms included “Acute Coronary Syndrome”, “No-Reflow Phenomenon”, “Slow-Flow Phenomenon”, “No Reflow”, “Cardiovascular Stroke”, “Chinese medicine injection”, “Traditional Chinese medicine”, “TCM injection”, “randomized controlled trial”, “RCT”, and “controlled clinical trial”, among others. As an example for PubMed, the search strategy is presented in Table 1.

**Table 1:** Pubmed retrieval strategy.

Procedure	Search query
#1	Acute Coronary Syndrome[Mesh] OR myocardial infarction[Title/Abstract] OR Infarction, Myocardial[Title/Abstract] OR Heart Attack[Title/Abstract] OR Myocardial Infarct[Title/Abstract] OR Cardiovascular Stroke[Title/Abstract] ORCoronary Syndrome, Acute[Title/Abstract] OR Syndrome, Acute Coronary[Title/Abstract] OR No-Reflow Phenomenon[Title/Abstract] OR Slow-Flow Phenomenon[Title/Abstract] OR No Reflow[Title/Abstract]
#2	Chinese medicine injection[Title/Abstract] OR Traditional Chinese medicine[Title/Abstract] OR TCM injection[Title/Abstract] OR Traditional Chinese medicine injection[Title/Abstract] OR chinese patent medicine injection[Title/Abstract] OR salvia ligustrazine injection[Title/Abstract] OR sheng mei injection[Title/Abstract] OR shenfu injection[Title/Abstract] OR blood plug tong injection[Title/Abstract] OR blood circulation injection[Title/Abstract] OR danhong injection[Title/Abstract] OR danshen injection[Title/Abstract] OR tanshinone injection[Title/Abstract] OR tanshinone iia sodium sulfonate injection[Title/Abstract] OR compound injection of red sage root[Title/Abstract] OR ixeris sonchifolia injection[Title/Abstract] OR pulse-activating injection[Title/Abstract] OR ginkgo leaf extract dipyridamole injection[Title/Abstract] OR salvia ligustrazine injection[Title/Abstract]
#3	Randomized controlled trial[Title/Abstract] OR RCT[Title/Abstract] OR controlled clinical trial[Title/Abstract] OR randomized[Title/Abstract] OR randomly[Title/Abstract] OR trial[Title/Abstract]
#4	#1 AND #2 AND #3

### Selection criteria

Studies were included if they met the inclusion criteria: (1) Eligible patients were those with acute coronary syndrome undergoing PCI; (2) The control group received standard treatments (conventional medical therapy according to contemporary clinical guidelines, typically including dual antiplatelet therapy [aspirin plus a P2Y₁₂ inhibitor (clopidogrel or ticagrelor)], statins, beta-blockers, angiotensin-converting enzyme inhibitors [ACEI] or angiotensin receptor blockers [ARB], and other necessary medications) alongside PCI. The experimental group received TCM injection formulations in addition to standard care, excluding studies that involved other TCM forms; (3) Key outcomes included the incidence of no-reflow or slow-reflow (TIMI flow grade ≤ II), Left Ventricular Ejection Fraction (LVEF), Left Ventricular End-Diastolic Dimension (LVEDD), Left Ventricular End-Systolic Diameter (LVESD), and the occurrence of Major Adverse Cardiovascular Events (MACE). The exclusion criteria were as follows: (1) Studies with fewer than 20 subjects per group to minimize potential bias from underpowered small-sample studies; (2) Studies from which key outcome data could not be extracted or calculated; (3) Patients with comorbidities such as end-stage renal disease, malignancies, or hematologic disorders; (4) Studies with unclear or inconsistent definitions of no-reflow; (5) Duplicately published studies; (6) Reviews, case reports, personal experience summaries, and meta-analyses; (7) Non-randomized controlled trials; (8) Publications in languages other than Chinese or English.

### Data extraction and risks of bias assessment

Quality of included studies was assessed using the Cochrane Risk of Bias 2.0 tool (RoB 2.0) (9). RoB 2.0 comprised five domains: bias arising from the randomization process, deviation from intended interventions, missing outcome data, measurement of outcomes, and selection of reported results. A comprehensive assessment was conducted after evaluating these five domains to determine the overall risk of bias, categorized as low, high, or unclear risk (10).

Data extraction was performed using a standardized form in Microsoft Excel. Extracted data included, but were not limited to: study characteristics (publication year, authors, journal name), participants (total sample size, age, and gender distribution), intervention details (interventions for experimental and control groups, route of administration, dosage, timing, and duration), outcome measures (definition, measurement methods, and units for each outcome), and results data (event counts and totals for binary variables, sample size, mean, and standard deviation for continuous variables). Two researchers independently completed the data extraction and cross-checked their findings; discrepancies were resolved by consensus or by consulting a third reviewer (J.Z.).

### Statistical analysis

In this network meta-analysis, various software and methods were employed to ensure the accuracy and visual representation of results. Core statistical analyses were performed using the Stata 18 software network package (11), which operates under a frequentist framework. Key indicators were calculated, and several significant visualizations were generated. The evidence network diagram intuitively displayed the relationships between different interventions, where dots represented interventions and their sizes indicated the sample sizes included; connecting lines represented direct comparisons between two interventions, with line thickness reflecting the number of studies using both interventions. The forest plot clearly illustrated the effect sizes and 95% confidence intervals (CIs) for each intervention. The surface under the cumulative ranking curve (SUCRA) was used to present the ranking of interventions (12), providing strong support for clinical decision-making. The funnel plot assessed publication bias to ensure the reliability of analytical results (13). The rank probability plot was created using the ggplot2 and ggalluvial packages in R 4.3.1 to display the probabilities of different interventions at specific ranking positions. A macro-enabled Excel file “RoB2_IRPG_beta_v9” was utilized to draw the risk of bias bar chart.

For statistics, odds ratios (ORs) and their 95% CIs were used to present results for the binary variables of no-reflow incidence and MACE incidence. The ORs effectively measured the impact of interventions on event probability, while the 95% CI provided a credible range for assessing result stability. For continuous variables such as LVEF, LVEDD, and LVESD, mean differences (MDs) served as effect analysis statistics. The MD intuitively reflected the average differences among interventions for these continuous variables, facilitating quantitative evaluation and comparison of intervention effects.

## Results

### Characteristics and quality evaluationof the included studies

**Figure 1:**
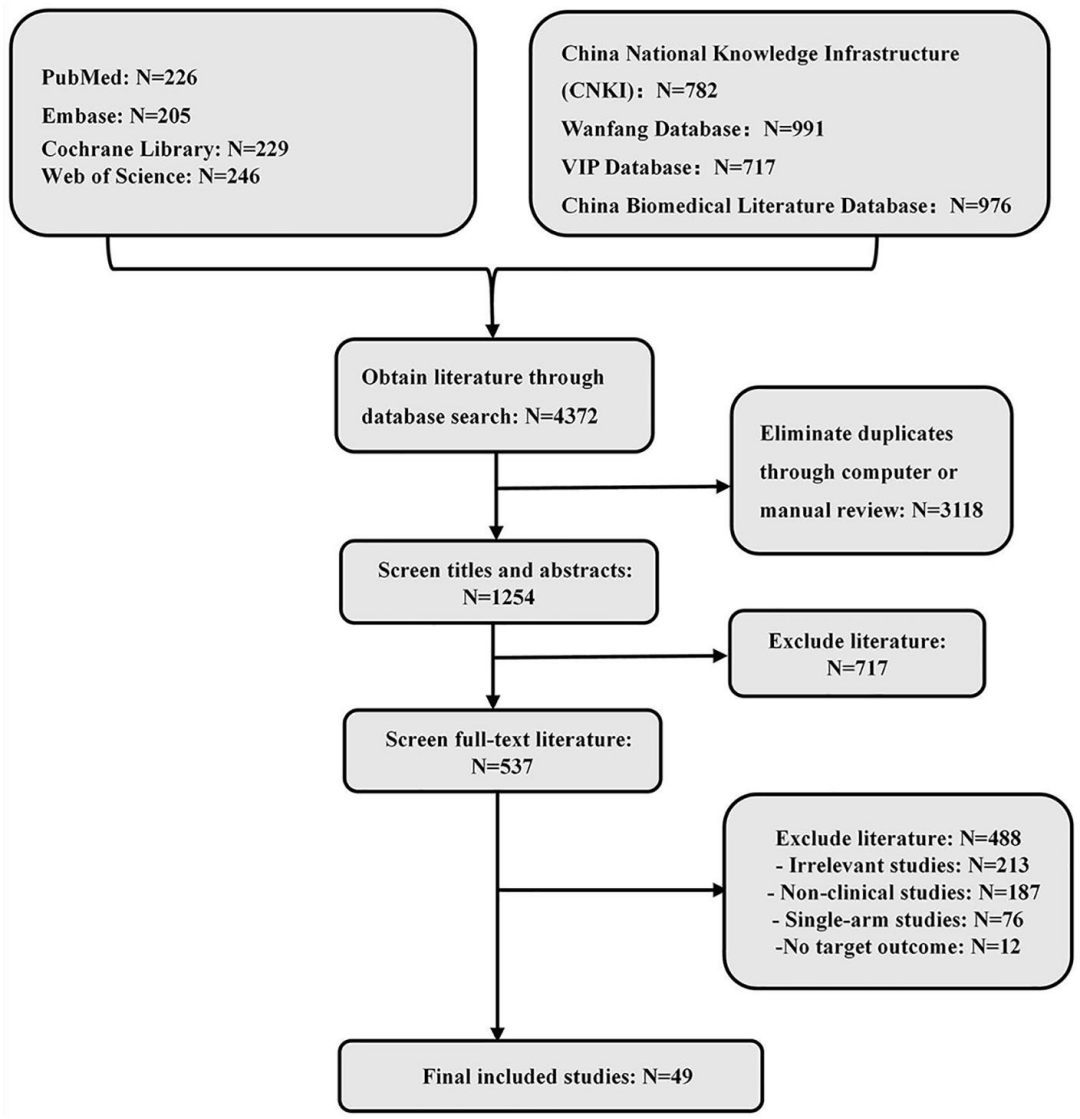
Literature screening flowchart.

A preliminary search identified 4,372 publications. After removing duplicates, 1,254 articles were screened. Following a review of titles and abstracts, 717 articles were excluded, leaving 537 for full-text review, ultimately resulting in the inclusion of 49 RCTs (14–62). The analysis included 5,535 patients and evaluated 14 different interventions: Shenfu injection, Shenmai injection, Shengmai injection, Danshenketo II A sulfonate injection, Danhong injection, Danshen injection, Shuxuetong injection, Danshenketo injection, Danshen polyphenol salt injection, Xuesaitong injection, Danshen Chuanxiong injection, Kudiezi injection, Ginkgo biloba injection, and standard treatment. The literature screening process is illustrated in Figure 1, and the basic characteristics of the included studies are detailed in Table 2.

**Table 2:** Baseline characteristics of the included studies.

Study (Author, Year https://www.ClinicalTrials.gov)	Sample size (male/female)		Mean age (Mean ± SD, year)		Intervention		Duration (day)	Outcomes
	Teatment group	Control group	Teatment group	Control group	Teatment group (Drug dosage)	Control group		
Xiao Wang 2024 NCT04493840	144 (121/23)	151 (125/26)	55.5	59.0	Shenfu injection 80 ml	Standard treatment	5 days	①②
Jiancheng Cao 2022 Not available	41 (25/16)	41 (22/19)	52.75 ± 4.36	54.12 ± 3.19	Ginseng injection 40 ml	Standard treatment	5 days	①⑤
Kai Xu 2022 Not available	93 (52/41)	93 (55/38)	66.56 ± 3.35	66.69 ± 3.78	Shengmai injection 60 ml	Standard treatment	7 days	①②③④⑤
Lu Yang 2021 Not available	100 (58/42)	100 (52/48)	58.06 ± 4.84	58.13 ± 5.16	Tanshinone IIA sodium sulfonate injection 80 mg	Standard treatment	7 days	①③④⑤
Qi Chen 2021 Not available	46 (29/17)	47 (27/20)	62.9 ± 9.5	63.2 ± 8.5	Danhong injection 40 ml	Standard treatment	–	①⑤
Lina Hao 2021 Not available	49 (22/27)	48 (20/28)	56.98 ± 4.02	57.47 ± 3.98	Shenfu injection 40 ml	Standard treatment	7 days	①⑤
Xiuhua Zhu 2020 Not available	70 (39/31)	70 (43/27)	65.07 ± 7.24	63.21 ± 6.63	Shenfu injection 60 ml	Standard treatment	10 days	①⑤
Bin Huang 2020 Not available	36 (24/12)	36 (23/13)	61.05 ± 3.64	62.74 ± 3.91	Shengmai injection 60 ml	Standard treatment	7 days	①②③④
Donglang Chen 2020 Not available	30 (17/13)	30 (16/14)	56.4 ± 5.2	56.8 ± 4.6	Danshen injection 20mL	Standard treatment	7 days	①
Mingyan Yao 2019 Not available	47 (28/19)	47 (30/17)	67.47 ± 7.99	67.61 ± 8.07	Shenfu injection 1 ml/kg	Standard treatment	–	① ⑤
Qi You 2019 ChiCTR1800019451	57 (48/9)	53 (47/6)	56.8 ± 8.9	55.4 ± 9.5	Danhong injection 40 ml	Standard treatment	7 days	①⑤
Jinghui Zhao 2019 Not available	60 (43/26)	60 (48/29)	68 ± 5	65 ± 7	Shuxuetong injection 8 ml	Standard treatment	7 days	①②③④⑤
Baixiang Yuan 2019 Not available	60 (38/22)	60 (41/19)	60.2 ± 5.5	60.9 ± 5.0	Danshen injection 30 ml	Standard treatment	3 days	①②③④⑤
Hongyan Ye 2019 Not available	39 (23/16)	39 (27/12)	59.27 ± 8.11	59.22 ± 8.39	Tanshinone injection 60 mg	Standard treatment	7 days	①②
Qi You 2019 ChiCTR1800019451	62 (53/9)	57 (50/7)	58.1 ± 9.9	58.0 ± 9.7	Danhong injection 40 ml	Standard treatment	7 days	①⑤
Yihui Zhang 2019 Not available	100 (57/43)	100 (59/41)	62.45 ± 7.42	61.93 ± 7.74	Shuxuetong injection 8 ml	Standard treatment	7 days	①②③⑤
Ying Zhang 2019 Not available	102 (52/50)	102 (50/52)	56 ± 5.3	54 ± 6.3	Tanshinone IIA sodium sulfonate injection 50 ml	Standard treatment	7 days	①⑤
Wenlu Chen 2018 Not available	45 (26/19)	45 (25/20)	53.06 ± 1.29	52.11 ± 1.09	Ginseng injection 40 ml	Standard treatment	5 days	①⑤
Hui Chang 2018 Not available	55 (37/18)	50 (31/19)	61.38 ± 4.09	59.67 ± 5.02	Danshen injection 20 ml	Danshen polyphenolic acid salt	3 days	①
Xuan Wang 2018 Not available	25 (20/5)	25 (15/10)	56.1 ± 5.3	55.8 ± 5.2	Danshen injection 20 ml	Standard treatment	7 days	①②
Li Qu 2018 Not available	58	54	–	–	Shenfu injection 60 ml	Standard treatment	10 days	①
Hongmin Zhou 2018 Not available	38 (25/13)	38 (21/17)	59.2 ± 5.4	58.7 ± 6.7	Danhong injection 40 ml	Standard treatment	10 days	①②⑤
Lianren Wang 2018 Not available	52 (31/21)	52 (28/24)	56.71 ± 6.25	57.29 ± 6.61	Xuesaitong injection 8 ml	Standard treatment	14 days	①②③
Dongmei Zhang 2018 Not available	60 (35/25)	60 (33/27)	62.97 ± 3.59	63.07 ± 3.60	Shenfu injection 80 ml	Standard treatment	–	①⑤
Shijing Na 2018 Not available	40 (22/18)	40 (24/16)	62.9 ± 8.5	63.7 ± 9.0	Shenfu injection 1 ml/kg	Standard treatment	–	①⑤
Li qin 2018 Not available	63 (29/34)	63 (33/30)	63.98 ± 1.25	63.41 ± 1.16	Danhong injection 20 ml	Standard treatment	7 days	①②③④⑤
Lin Zhou 2017 Not available	70	60	75 ± 9	74 ± 8	Salvia ligustrazine injection 10 ml	Standard treatment	7 days	①⑤
Dongmei Zhang 2017 Not available	40 (22/18)	40 (25/15)	61.6 ± 11.5	63.2 ± 11.2	Shenfu injection 80 ml	Standard treatment	–	①⑤
Jing Wu 2017 Not available	44	36	–	–	Danhong injection 20 ml	Standard treatment	–	①
Yujuan Wang 2017 Not available	62 (35/27)	62 (38/24)	58.4 ± 9.6	57.6 ± 10.1	Ixeris sonchifolia injection 40 ml	Standard treatment	14 days	①
Wei Leng 2016 Not available	120 (80/40)	120 (77/43)	56.9 ± 10.7	58.1 ± 11.2	Danhong injection 20 ml	Standard treatment	–	①
Qiangfu Wu 2015 Not available	40	40	–	–	Shuxuetong injection 8 ml	Standard treatment	7 days	①②③④⑤
Junpeng Feng 2015 Not available	34 (17/17)	36 (18/18)	62.5 ± 7.4	60.8 ± 10.0	Xuesaitong injection 400 mg	Standard treatment	14 days	①
Min Jia 2015 Not available	60 (35/25)	60 (40/20)	62.23 ± 11.26	64.56 ± 12.85	Danhong injection 20 ml	Standard treatment	–	①
Hanying Zhou 2015 Not available	39 (27/12)	40 (31/9)	65 ± 11	62 ± 11	Shuxuetong injection 8 ml	Standard treatment	7 days	①②③④⑤
Xiang Zhou 2014 Not available	100 (71/29)	100 (75/25)	55.9 ± 1.48	56.0 ± 1.51	Salvia ligustrazine injection 10 ml	Standard treatment	–	①⑤
Sheng Guo 2014 Not available	39 (26/13)	39	55.6 ± 11.8	–	Ginseng injection 50 ml	Standard treatment	7 days	①
Xiaozheng Yang 2014 Not available	30 (19/11)	30 (17/13)	57.77 ± 10.70	57.93 ± 10.37	Ginseng injection 5 ml	Standard treatment	3 days	①⑤
Xiaozheng Yang 2014-2 Not available	25 (17/8)	25 (18/7)	57.44 ± 11.09	59.40 ± 11.00	Ginseng injection 30 ml	Standard treatment	–	①⑤
Jing Wang, 2014 Not available	138 (68/70)	136 (66/70)	59.32 ± 4.62	60.61 ± 4.61	Salvia ligustrazine injection 40 ml	Standard treatment	14 days	①⑤
Xuguang Feng, 2013 Not available	60 (32/28)	36 (20/16)	62.5 ± 10.1	61.6 ± 11.3	Shuxuetong injection 6 ml	Standard treatment	14 days	①
Baizhi Wang, 2013 Not available	30 (23/7)	30 (21/9)	65.22 ± 7.54	63.61 ± 8.21	Danhong injection 20 ml	Standard treatment	–	①
Rui Wang, 2011 Not available	36 (27/12)	37 (22/15)	–	–	Salvia ligustrazine injection 10 ml	Standard treatment	7–10 days	①
Deqiang Zhao, 2010 Not available	37 (29/8)	37 (25/12)	66.4 ± 10.4	66.2 ± 12.0	Ginkgo damo injection 20 ml	Standard treatment	7 days	①⑤
Xuan Wang, 2010 Not available	32 (19/13)	30 (16/14)	58.0 ± 14.9	54.9 ± 15.2	shengmai injection 50 ml	Standard treatment	7 days	①②③④
Weiguo Shi, 2010 Not available	55	51	–	–	Danhong injection 40 ml	Standard treatment	10 days	①
Xiaofei Wang, 2008 Not available	30 (20/10)	30 (24/6)	54.0 ± 4.9	54.9 ± 5.2	Shengmai injection 50 ml	Standard treatment	7 days	①②③④
Jinsong He, 2006 Not available	68 (36/32)	64 (30/34)	61.18 ± 12.13	60.13 ± 11.15	Shenfu injection 40 ml	Standard treatment	7 days	①
Guohai Su, 2005 Not available	47 (29/18)	46 (30/16)	61.8 ± 12.3	60.3 ± 11.5	Shenfu injection 40 ml	Standard treatment	7 days	①

Secondary endpoints: ① Incidence of no-reflow or slow-flow ② Left ventricular ejection fraction ③ Left ventricular end-diastolic internal diameter ④ Left ventricular end-systolic internal diameter ⑤ Incidence of major adverse cardiovascular events. Standard Treatment: dual antiplatelet therapy (aspirin plus a P2Y₁₂ inhibitor, either ticagrelor or clopidogrel), statins, beta-blockers, angiotensin-converting enzyme inhibitors (ACEI) or angiotensin receptor blockers (ARB), and other necessary medications per contemporary practice guidelines.

**Figure 2:**
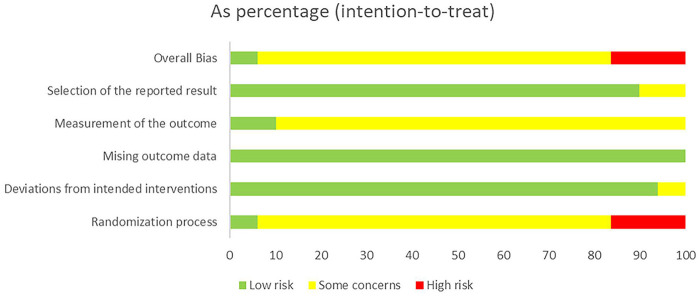
Risk of bias assessment.

The risk of bias assessment for the 49 included studies revealed considerable variation in methodological quality. Of these, 41 studies explicitly employed random allocation methods, while the remaining 8 either allocated by treatment sequence or did not describe their allocation methods, resulting in a high risk rating for these studies in terms of randomization. Only 3 studies reported blinding of both participants and investigators, while the others did not mention the use of blinding. Additionally, 44 studies did not disclose whether outcome assessors were aware of the interventions received by the subjects, indicating potential risk. A more detailed presentation and analysis of the bias risk can be found in Figure 2.

### Network meta-analysis in acute coronary syndrome with no-reflow or slow flow after PCI

**Figure 3:**
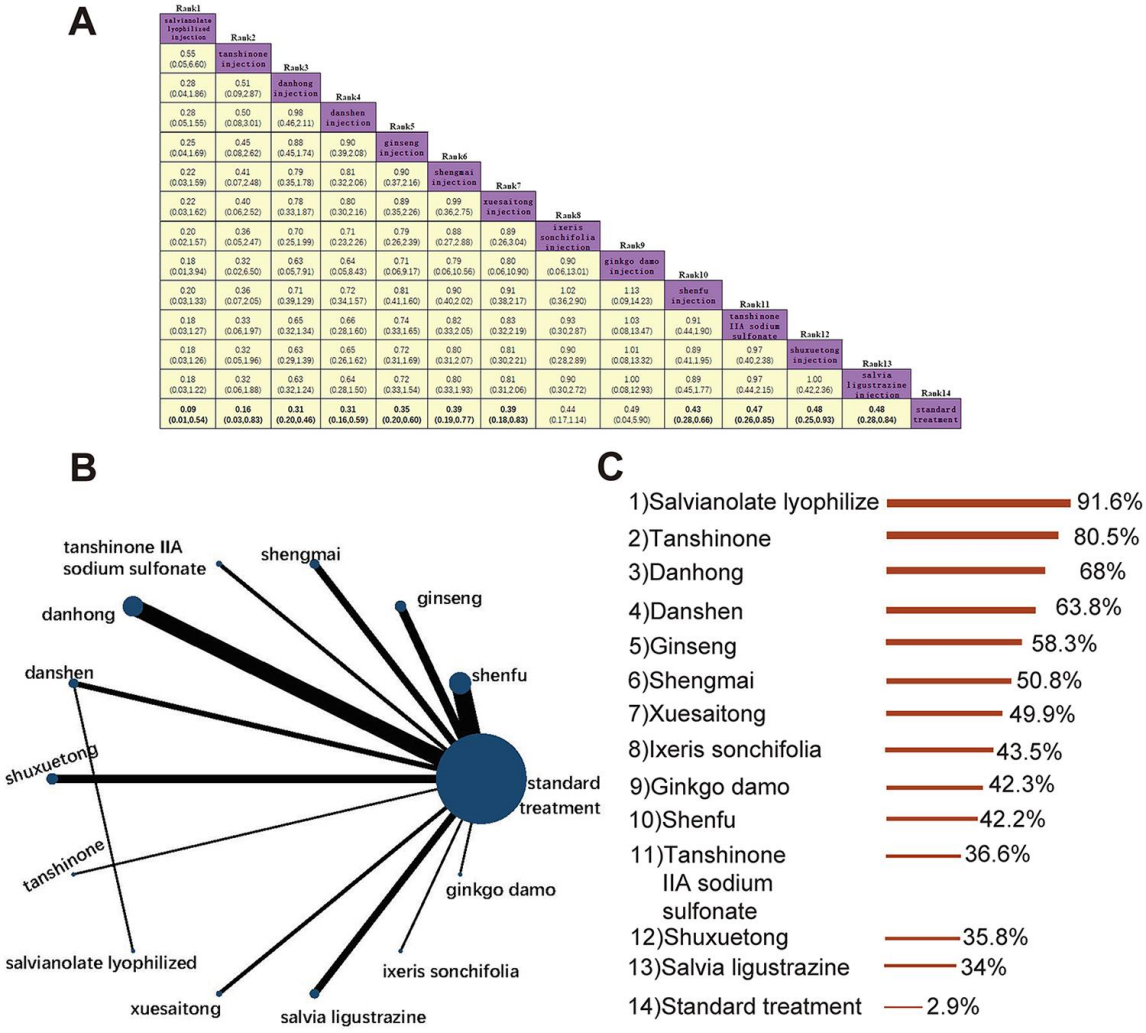
Incidence of no-flow or slow flow. **(A)** Pairwise comparisons of the incidence of no-reflow or slow flow. **(B)** Evidence network diagram of no-flow or slow flow incidence. **(C)** Treatment rankings (SUCRA).

A total of 49 studies evaluated the incidence of no-flow or slow flow, comparing 13 traditional Chinese medicine (TCM) injections (Shenfu, Ginseng, Shengmai, Tanshinone IIA, Danhong, Danshen, Shuxuetong, Tanshinone, Salvianolate, Xuesaitong, Salvia ligustrazine, Ixeris sonchifolia, and Ginkgo damo) with standard treatment. The evidence network is shown in Figure 3B. No closed loops were formed, eliminating the need for inconsistency testing. The results demonstrated that Salvianolate, Tanshinone, Danhong, Danshen, Ginseng, Shengmai, Xuesaitong, Shenfu, Tanshinone IIA, Shuxuetong, and Salvia ligustrazine significantly reduced the incidence of no-flow or slow flow compared to standard treatment, with odds ratios (ORs) ranging from 0.09 to 0.48. In contrast, Ginkgo damo and Ixeris sonchifolia showed no significant benefit (Figure 3A). Based on SUCRA rankings, Salvianolate was identified as the most effective TCM injection for reducing no-flow or slow flow incidence, followed by Tanshinone and Danhong. Standard treatment ranked the lowest in terms of efficacy (Figure 3C).

**Figure 4:**
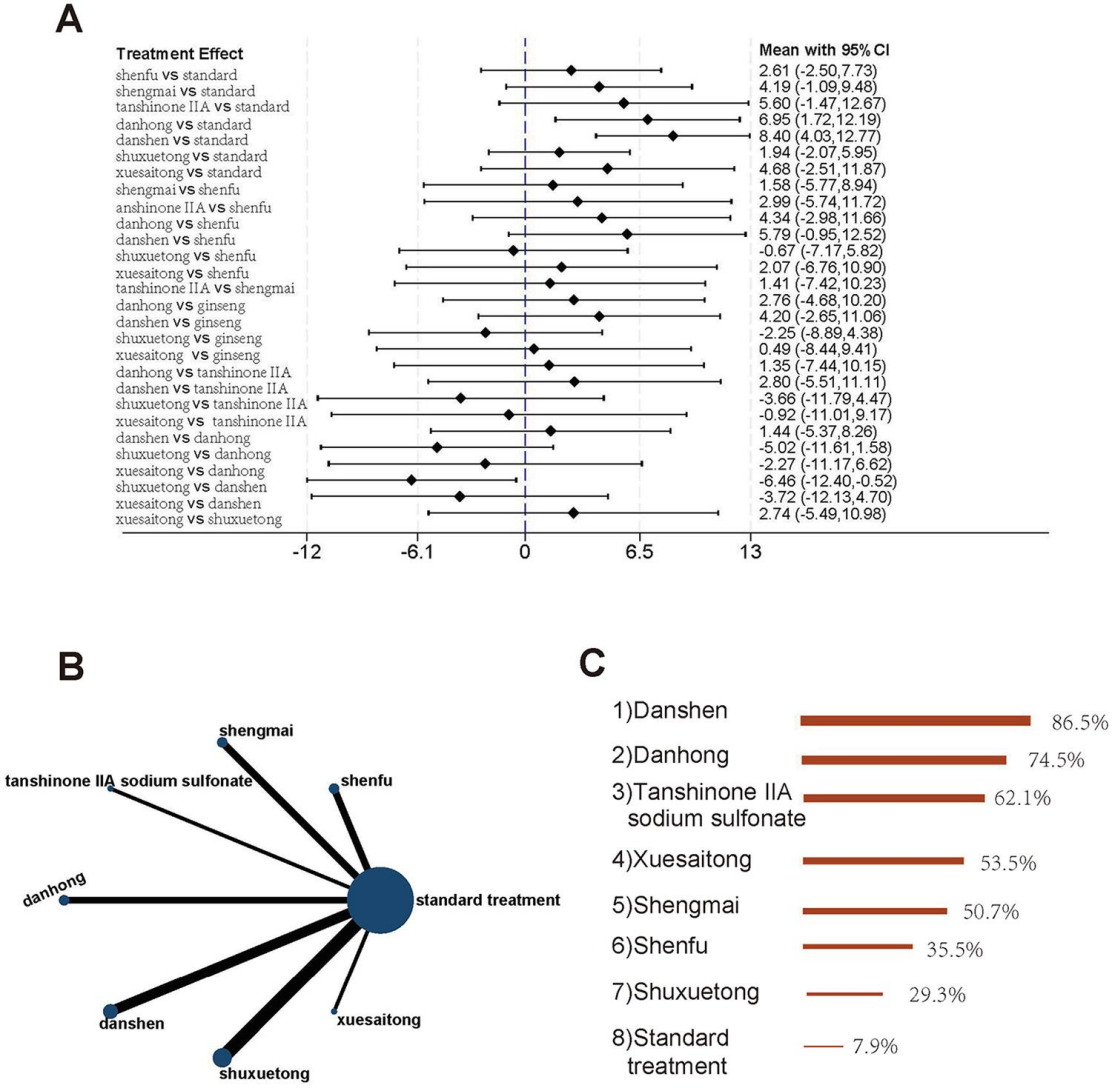
Studies involving LVEF **(A)** forest plot of pairwise comparisons for LVEF. **(B)** Evidence network diagram of LVEF. **(C)** Treatment rankings (SUCRA).

A total of 15 studies evaluated LVEF outcomes, comparing standard treatment with Shenfu, Shengmai, Tanshinone IIA sodium sulfonate, Danhong, Danshen, Shuxuetong, and Xuesaitong injections (8 interventions in total). The evidence network is shown in Figure 4B. The results demonstrated that Danshen injection [MD = 8.40, 95% CI (4.03, 12.77)] and Danhong injection [MD = 6.95, 95% CI (1.72, 12.19)] significantly improved LVEF compared to standard treatment. Additionally, Danshen injection showed superior efficacy over Shuxuetong injection [MD = 6.46, 95% CI (0.52, 12.40)]. Other TCM injections did not exhibit significant advantages over standard treatment (Figure 4A). According to SUCRA rankings, Danshen injection was identified as the most effective intervention for improving LVEF, followed by Danhong injection and Tanshinone IIA sodium sulfonate injection. Standard treatment ranked the lowest in terms of efficacy (Figure 4C).

**Figure 5:**
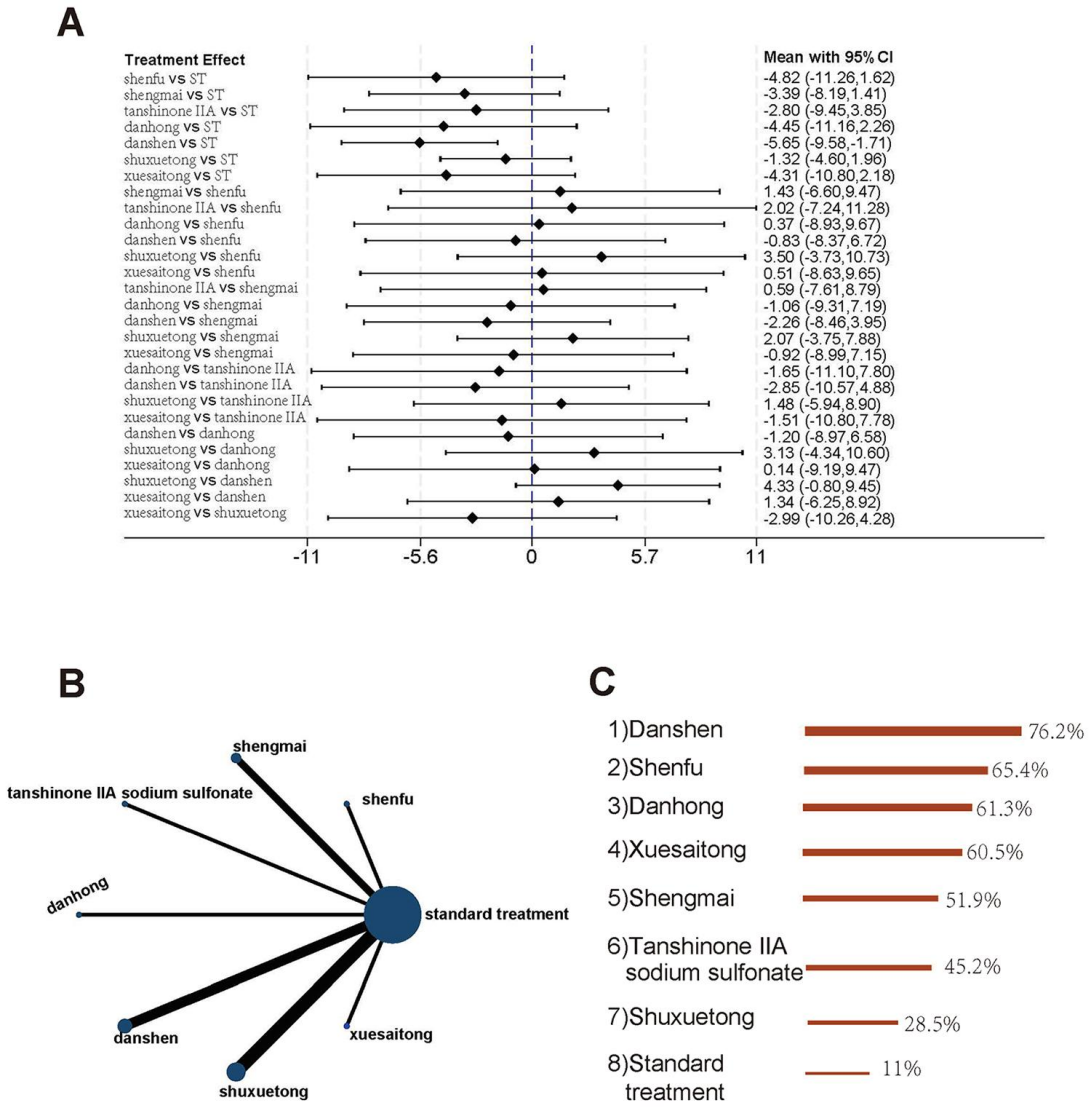
Studies involving LVEDD. **(A)** Forest plot of pairwise comparisons for LVEDD. **(B)** Evidence network diagram of LVEDD. **(C)** Treatment rankings (SUCRA).

A total of 13 studies evaluated LVEDD outcomes, comparing standard treatment with Shenfu, Shengmai, Tanshinone IIA sodium sulfonate, Danhong, Danshen, Shuxuetong, and Xuesaitong injections (8 interventions in total). The evidence network is shown in Figure 5B. The analysis revealed that only Danshen injection [MD = −5.65, 95% CI (−9.58, −1.71)] combined with standard treatment significantly reduced LVEDD compared to standard treatment alone. Other TCM injections did not demonstrate a statistically significant advantage over standard treatment, and no notable differences were observed among the various TCM interventions (Figure 5A). According to SUCRA rankings, Danshen injection emerged as the most effective intervention for reducing LVEDD, followed by Shenfu injection and Danhong injection. Standard treatment ranked the lowest in terms of efficacy (Figure 5C).

**Figure 6:**
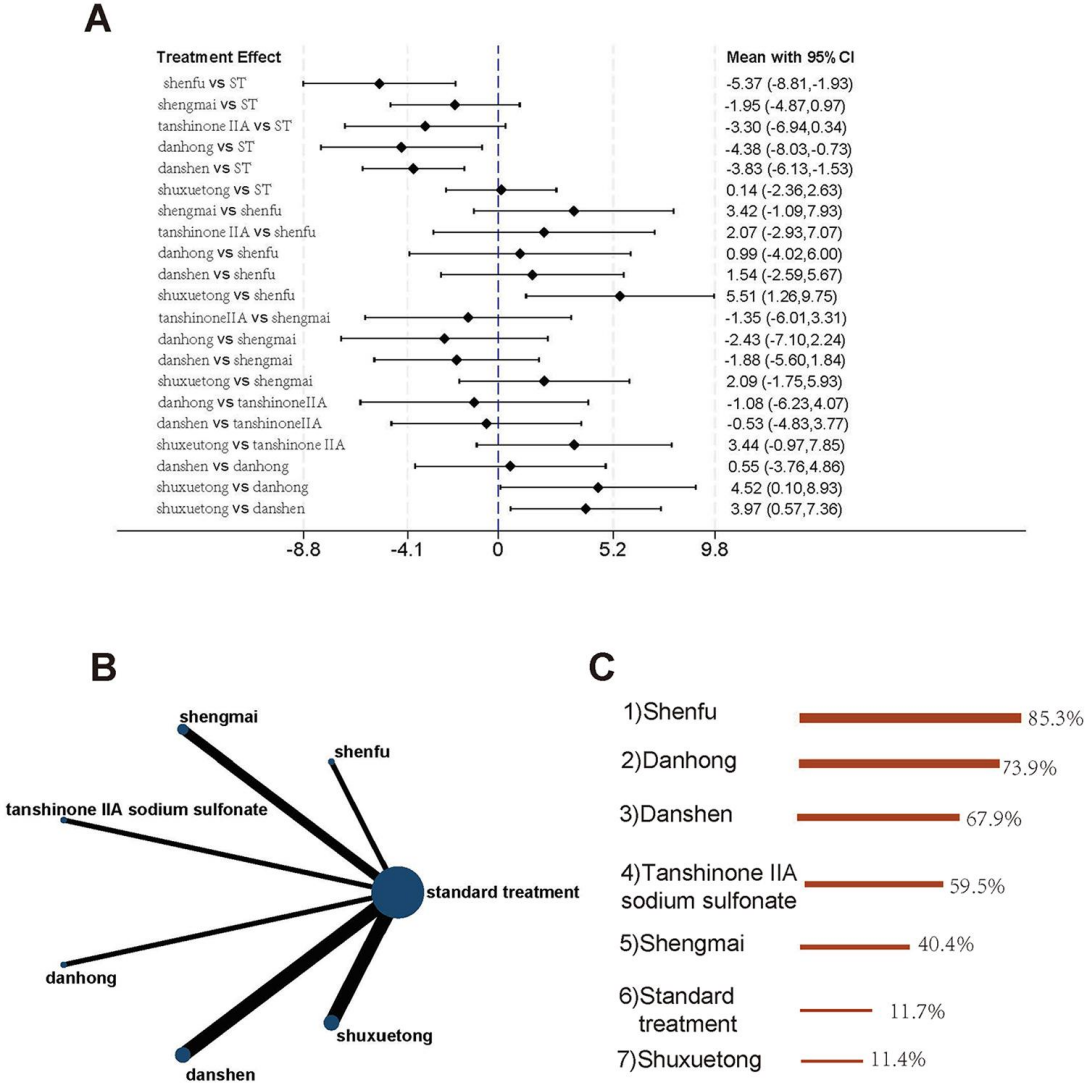
Studies involving LVESD **(A)** forest plot of pairwise comparisons for LVESD. **(B)** Evidence network diagram of LVESD. **(C)** Treatment rankings (SUCRA).

A total of 11 studies involving 1,149 patients evaluated LVESD outcomes, comparing standard treatment with Shenfu, Shengmai, Tanshinone IIA sodium sulfonate, Danhong, Danshen, and Shuxuetong injections (7 interventions in total). The evidence network is shown in Figure 6B. The results demonstrated that Shenfu injection [MD = −5.37, 95% CI (−8.81, −1.93)], Danhong injection [MD = −4.38, 95% CI (−8.03, −0.73)], and Danshen injection [MD = −3.83, 95% CI (−6.13, −1.53)] significantly reduced LVESD compared to standard treatment. Furthermore, Shenfu injection [MD = −5.51, 95% CI (−9.75, −1.26)], Danhong injection [MD = −4.52, 95% CI (−8.93, −0.10)], and Danshen injection [MD = −3.97, 95% CI (−7.36, −0.57)] also showed superior efficacy over Shuxuetong injection, with statistically significant differences (Figure 6A). According to SUCRA rankings, Shenfu injection was identified as the most effective intervention for reducing LVESD, followed by Danhong injection and Danshen injection. Standard treatment ranked the lowest in terms of efficacy (Figure 6C).

**Figure 7:**
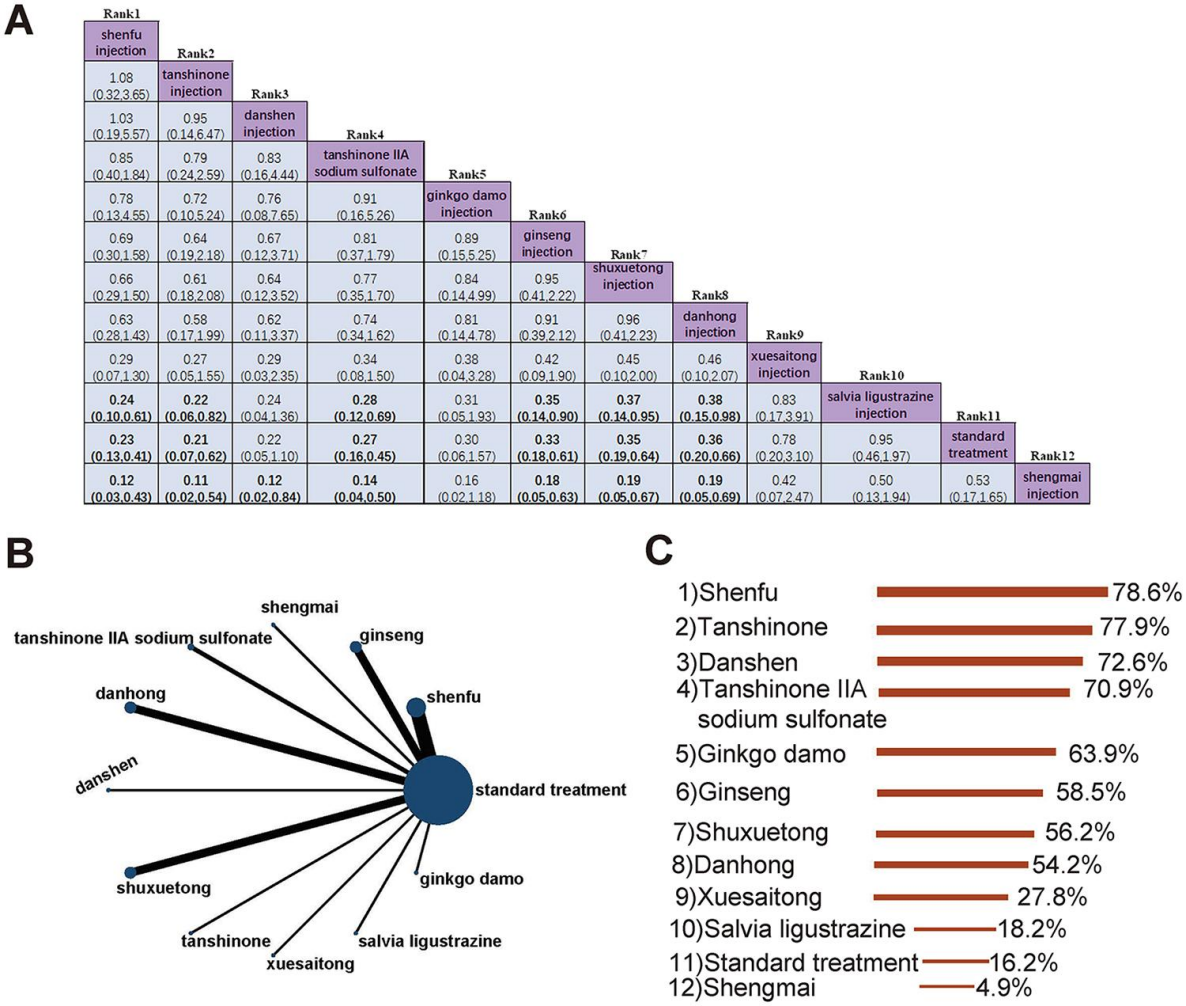
The incidence of MACE. **(A)** Pairwise comparisons of the incidence of MACE. **(B)** Evidence network diagram of MACE. **(C)** Treatment rankings (SUCRA).

A total of 27 studies involving 3,368 participants evaluated MACE outcomes, comparing standard treatment with Shenfu, Ginseng, Shengmai, Tanshinone IIA sodium sulfonate, Danhong, Danshen, Shuxuetong, Tanshinone, Xuesaitong, Salvia ligustrazine, and Ginkgo damo injections (12 interventions in total). The evidence network is shown in Figure 7B. The results demonstrated that Shenfu injection [OR = 0.23, 95% CI (0.13, 0.43)], Tanshinone injection [OR = 0.21, 95% CI (0.07, 0.62)], Tanshinone IIA sodium sulfonate injection [OR = 0.27, 95% CI (0.16, 0.45)], Ginseng injection [OR = 0.33, 95% CI (0.18, 0.61)], Shuxuetong injection [OR = 0.35, 95% CI (0.19, 0.64)], and Danhong injection [OR = 0.36, 95% CI (0.20, 0.66)] significantly reduced the incidence of MACE compared to standard treatment. Additionally, these injections also showed superior efficacy over Salvia ligustrazine injection and Shengmai injection (Figure 7A). According to SUCRA rankings, Shenfu injection was identified as the most effective intervention for reducing MACE incidence, followed by Tanshinone injection and Danshen injection. Standard treatment ranked the lowest in terms of efficacy (Figure 7C).

In summary, by synthesizing the findings across all efficacy and safety endpoints, distinct profiles for the TCM injections emerge. Interventions such as Danhong Injection, Shenfu Injection, and Danshen Injection consistently demonstrated broad efficacy, appearing in the top ranks for multiple critical outcomes including no-reflow incidence, cardiac function (LVEF, LVESD), and MACE reduction. These can be considered “all-rounders” offering comprehensive benefits. In contrast, other injections showed more specialized effects; for instance, Salvianolate Lyophilized Injection was ranked highest specifically for preventing no-reflow, while Tanshinone Injection was particularly effective for MACE reduction. This synthesis provides clinicians with a practical hierarchy: for broad-spectrum improvement, Danhong, Shenfu, or Danshen are prominent choices, whereas for targeting a specific predominant concern like no-reflow, Salvianolate may be the optimal specialist agent.

### Publication bias

**Figure 8:**
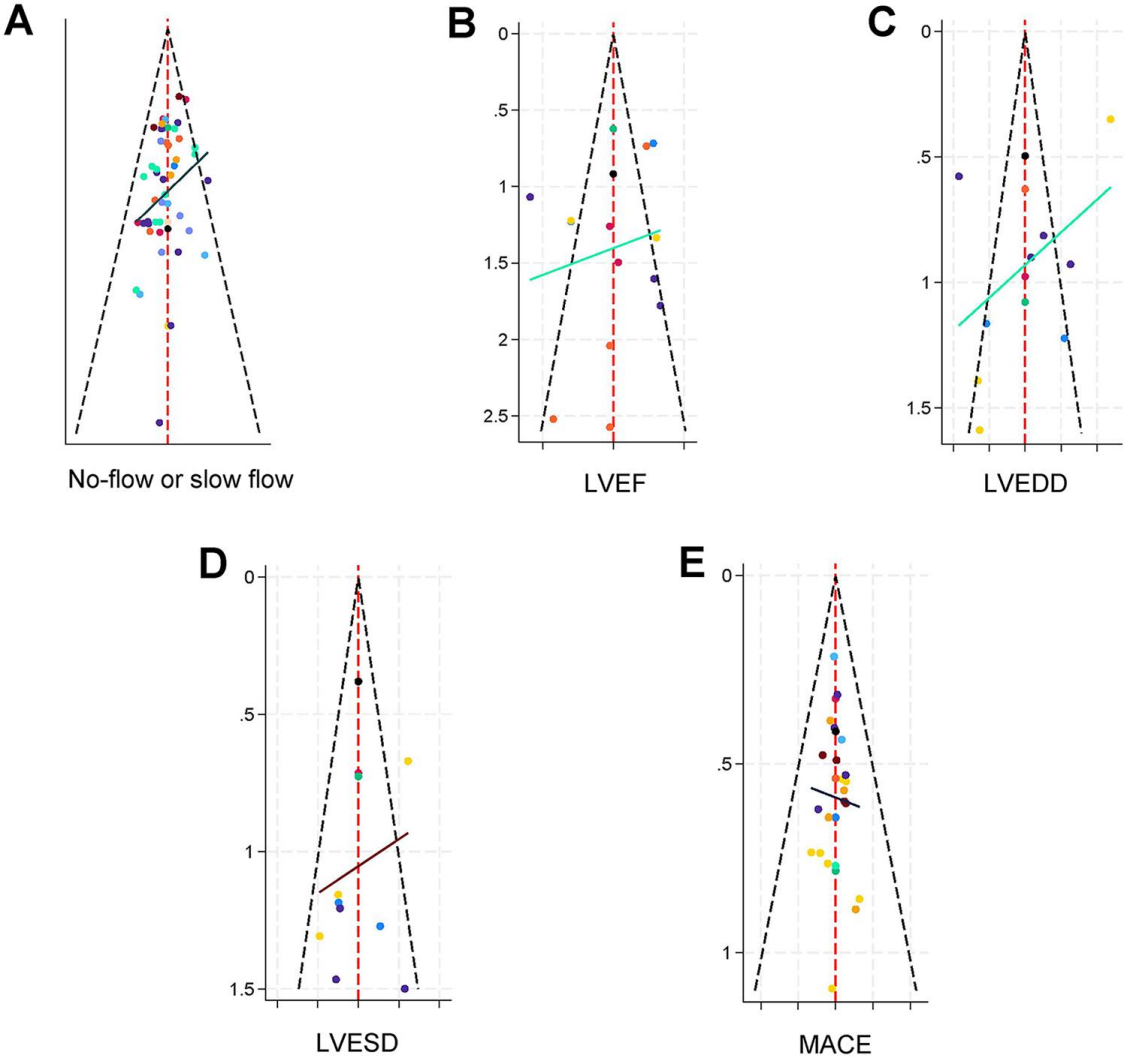
Funnel Plots for the Five Outcome Indicators. **(A)** No-reflow incidence. **(B)** Left ventricular ejection fraction (LVEF). **(C)** Left ventricular end-diastolic diameter (LVEDD). **(D)** Left ventricular end-systolic diameter (LVESD). **(E)** Major adverse cardiovascular events (MACE). Plots **A**, **D**, and **E**, with all or most data points within the 95% confidence intervals, indicate low heterogeneity. In contrast, plots **B** (LVEF) and **C** (LVEDD), showing a greater number of points outside the confidence intervals, suggest significant heterogeneity among the included studies).

In this study, five outcome indicators were set, with each indicator including more than 10 studies. By analyzing these data through funnel plots, we observed the distribution of the effect sizes on both sides of each study. The funnel plots of the outcomes can be seen in Figure 8. Specifically, in Figure 8E, all study points are neatly contained within the 95% confidence interval, with no points falling outside this range. Figures 8A,D show a similar situation, with only one point lying outside the 95% confidence interval. This indicates low heterogeneity among the studies assessing the outcomes of no-reflow, LVESD, and MACE. In other words, these studies yielded consistent results when evaluating the above outcome indicators, and there was little difference between them, which is beneficial for comprehensive analysis and interpretation of these indicators.

However, Figures 8B,C show a different scenario. In these two funnel plots, a relatively larger number of study points fall outside the 95% confidence interval. This suggests that there is significant heterogeneity among the studies with LVEF and LVEDD as outcome indicators. In other words, different studies yielded markedly different results when assessing LVEF and LVEDD. This larger heterogeneity may be due to various factors, such as differences in the study populations included, the measurement methods used, and the specific implementation details of the interventions. These variations could lead to a greater degree of dispersion in the results for the LVEF and LVEDD indicators, increasing the difficulty of conducting a comprehensive analysis and interpretation of the findings related to these indicators.

## Discussion

This network meta-analysis comprehensively evaluated the efficacy and safety of 13 TCM injections for ACS patients with no-reflow or slow flow after PCI. The most significant finding is that TCM injections, as adjuncts to standard therapy, are broadly effective, but they can be categorized into distinct efficacy profiles. Specifically, interventions like Danhong Injection, Shenfu Injection, and Danshen Injection emerged as consistent top performers across multiple endpoints, while others, such as Salvianolate Lyophilized Injection, demonstrated highly specific efficacy. This synthesis provides a practical, evidence-based hierarchy to guide clinical selection, moving beyond a one-size-fits-all approach.

A key insight from our analysis is the distinction between “all-rounder” and “specialist” interventions. Danhong Injection exemplified the “all-rounder” profile, showing significant benefits for improving no-reflow incidence, LVEF, LVESD, and reducing MACE. Similarly, Shenfu Injection ranked best for reducing LVESD and MACE, and Danshen Injection for improving LVEF and LVEDD, indicating their broad-spectrum cardioprotective effects. In contrast, Salvianolate Lyophilized Injection served as a “specialist”, ranking highest specifically for preventing the no-reflow phenomenon itself. This pattern suggests that the choice of TCM injection can be tailored to the patient's most pressing clinical need.

The clinical context of no-reflow phenomenon warrants emphasis. No-reflow or slow flow following PCI in ACS patients refers to impaired myocardial perfusion after successful revascularization, significantly increasing the risk of major cardiovascular events and mortality (3, 4). Current management strategies include vasodilators like nicorandil (63), antiplatelet agents such as ticagrelor (64), calcium channel blockers (e.g., verapamil (65), glycoprotein IIb/IIIa inhibitors like tirofiban (66), and non-pharmacological approaches including thrombus aspiration (67) and delayed stenting (68). Despite these available treatments, there remains substantial need for more effective interventions to address postoperative cardiac recovery limitations.

The distinct efficacy profiles observed in our analysis are supported by established pharmacological mechanisms. Danhong Injection's multi-target effects are attributed to its active components including danshenone, salvianolic acid, and hydroxy safflower yellow A, which collectively provide antioxidant, anti-inflammatory, antiplatelet aggregation, and anti-apoptotic benefits (69, 70). Shenfu Injection mediates mitochondrial autophagy to protect against ischemia-reperfusion injury through the HIF-1α/BNIP3 pathway (71), while Danshen Injection's tanshinones and salvianolic acids contribute to its anticoagulation, anti-inflammatory, and antioxidant properties (72). Salvianolate Lyophilized Injection exerts its specialized effects through potent microcirculation improvement and vascular endothelial protection (73, 74), directly addressing the core pathophysiology of no-reflow.

Our findings should be interpreted considering several limitations. First, the overall high risk of bias in many included studies, particularly concerning blinding, may have led to an overestimation of the treatment effects (9, 10). Second, the considerable heterogeneity observed in some outcomes (e.g., LVEF and LVEDD) likely stems from variations in patient demographics, PCI procedures, and TCM injection dosages, reducing the precision of these estimates. Third, the notable disparity in sample sizes across interventions means that rankings for less-studied injections (e.g., Salvianolate) should be considered exploratory, whereas those for extensively studied ones (e.g., Danhong) are more robust. Fourth, the lack of TCM syndrome differentiation in included studies limits our understanding of whether certain injections are more effective for specific patient subtypes. Finally, all participants were Chinese, potentially affecting the generalizability of our findings to other populations.

Notwithstanding these limitations, our study provides crucial evidence for personalizing TCM injection selection. For clinicians seeking comprehensive protection, Danhong Injection represents the most versatile choice. When targeting specific pathophysiological processes, Salvianolate Lyophilized Injection may be optimal for no-reflow prevention, Shenfu Injection for managing ventricular remodeling and MACE, and Danshen Injection for enhancing cardiac systolic function. Future research should prioritize large-scale, double-blind RCTs that directly compare these promising injections, incorporate TCM syndrome differentiation, explore potential synergistic combinations, and validate these findings in diverse ethnic populations.

## Data Availability

The original contributions presented in the study are included in the article/Supplementary Material, further inquiries can be directed to the corresponding author.
