# Supplementary figures and images for: Efficacy and safety of Traditional Chinese Medicine injections for no-reflow or slow flow in patients with acute coronary syndrome after percutaneous coronary intervention: a systematic review and network meta-analysis
Source: Front Cardiovasc Med. 2026 Jan 7;12:1619345. doi: 10.3389/fcvm.2025.1619345 (PMC12819830; doi:10.3389/fcvm.2025.1619345)

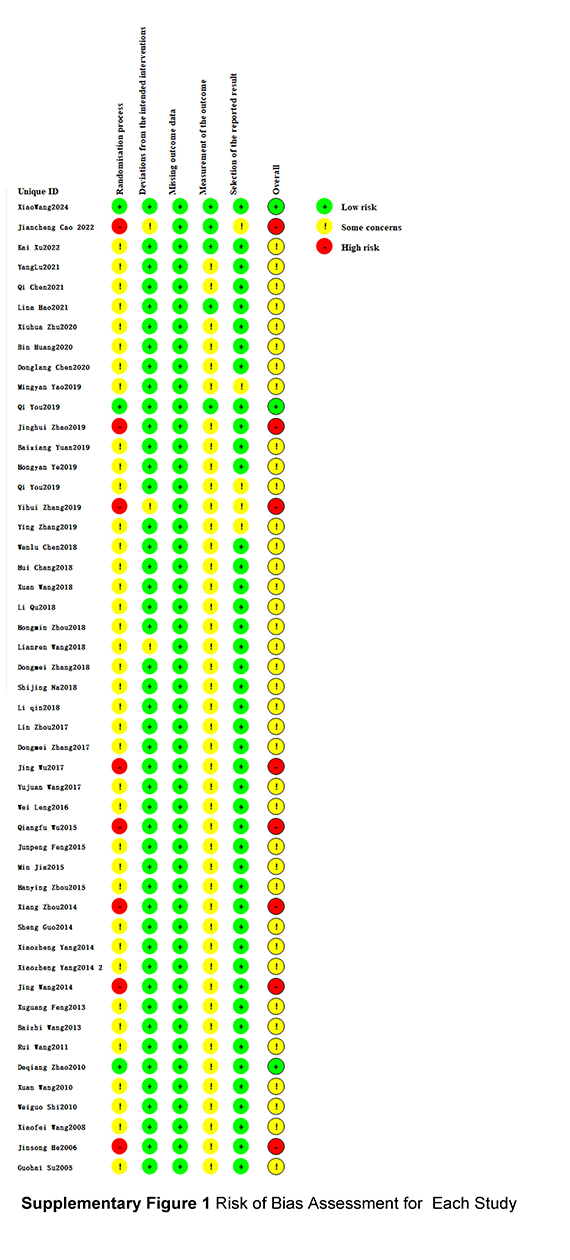

Supplement: Supplementary file 1 [file Image1.tif]

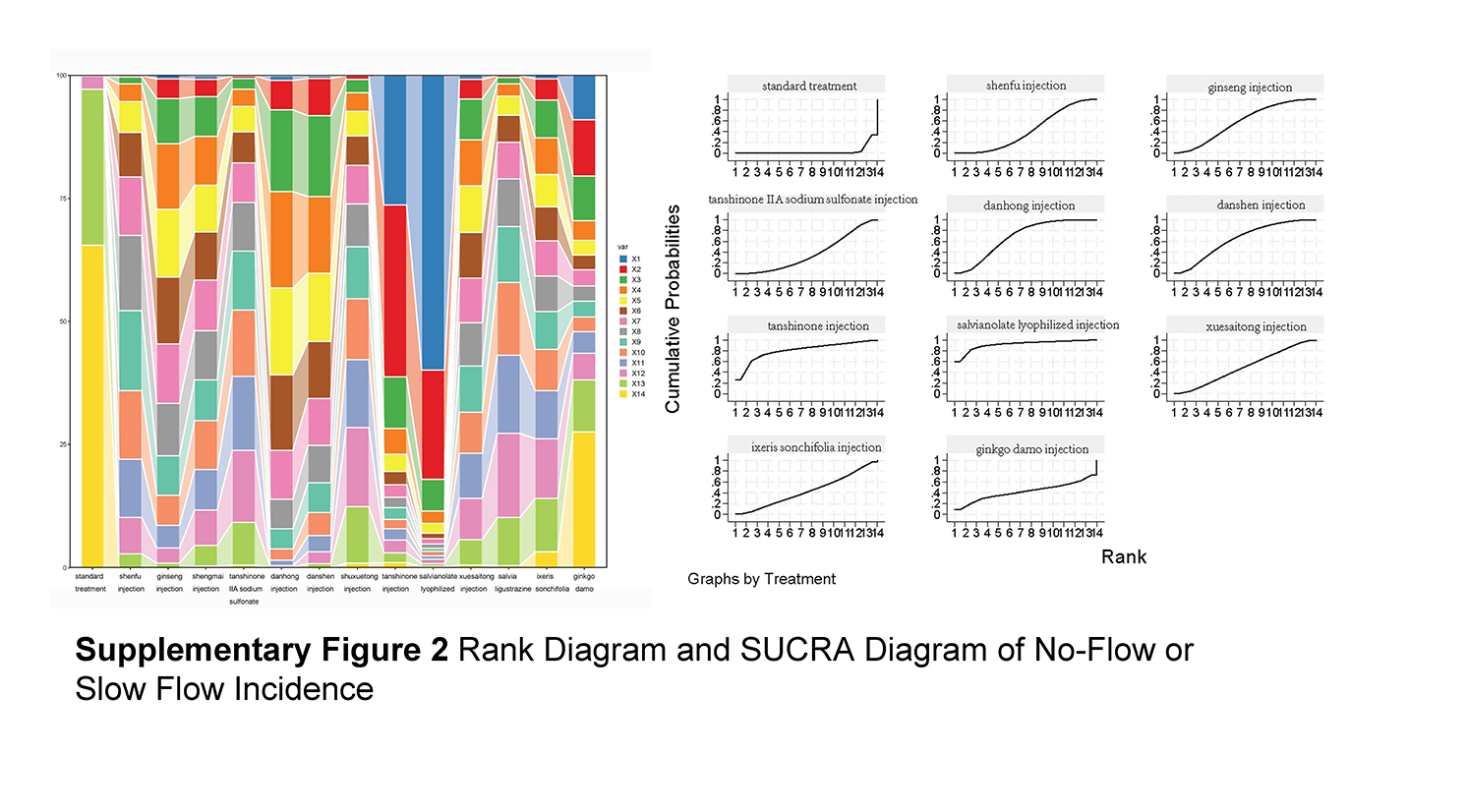

Supplement: Supplementary file 2 [file Image2.tif]

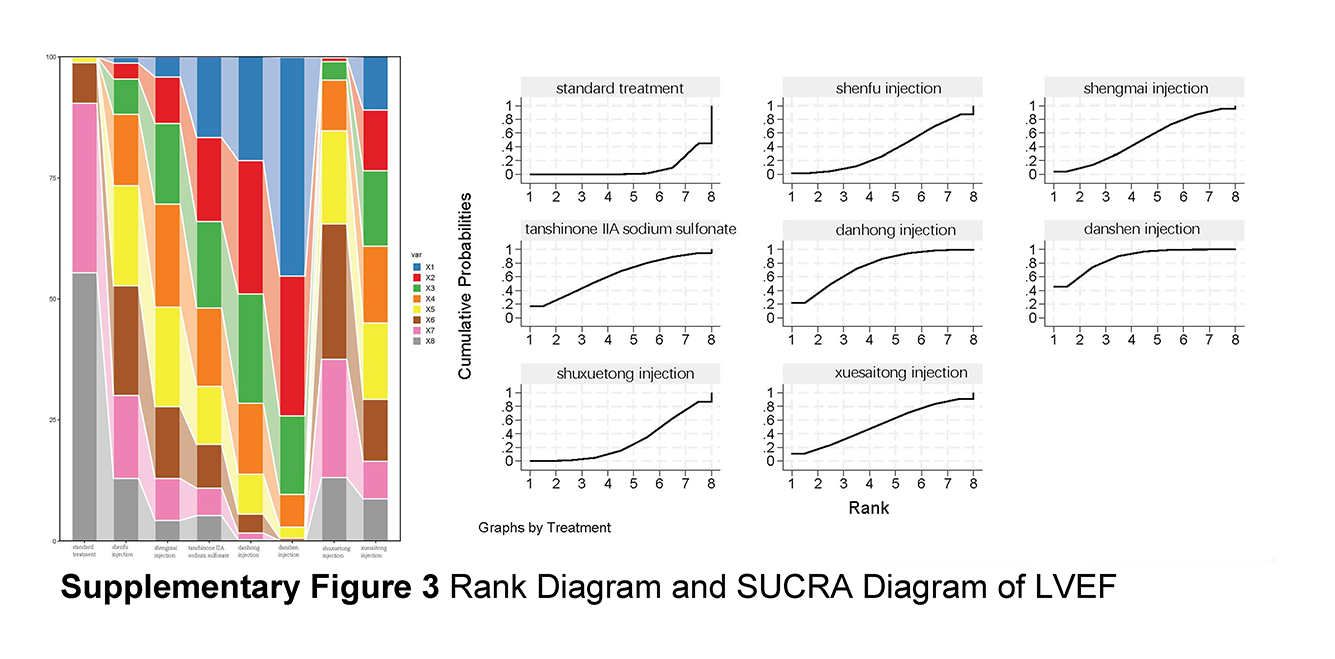

Supplement: Supplementary file 3 [file Image3.tif]

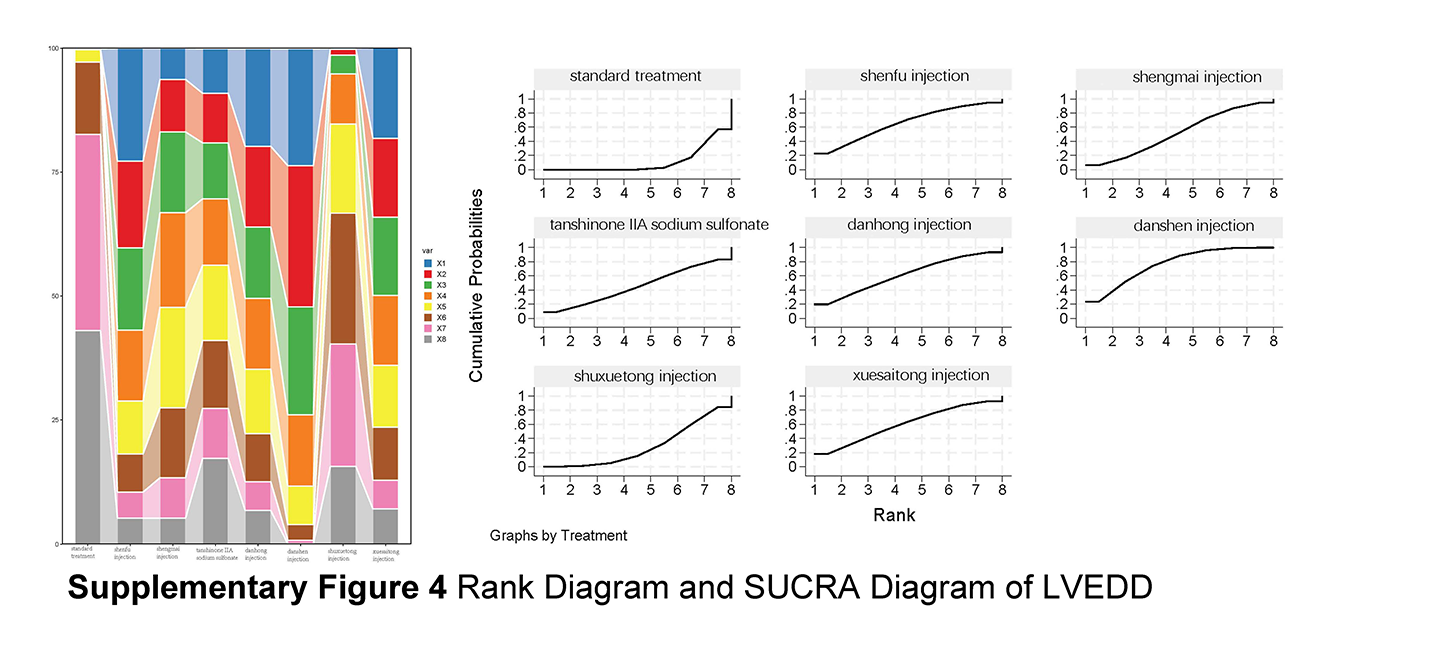

Supplement: Supplementary file 4 [file Image4.tif]

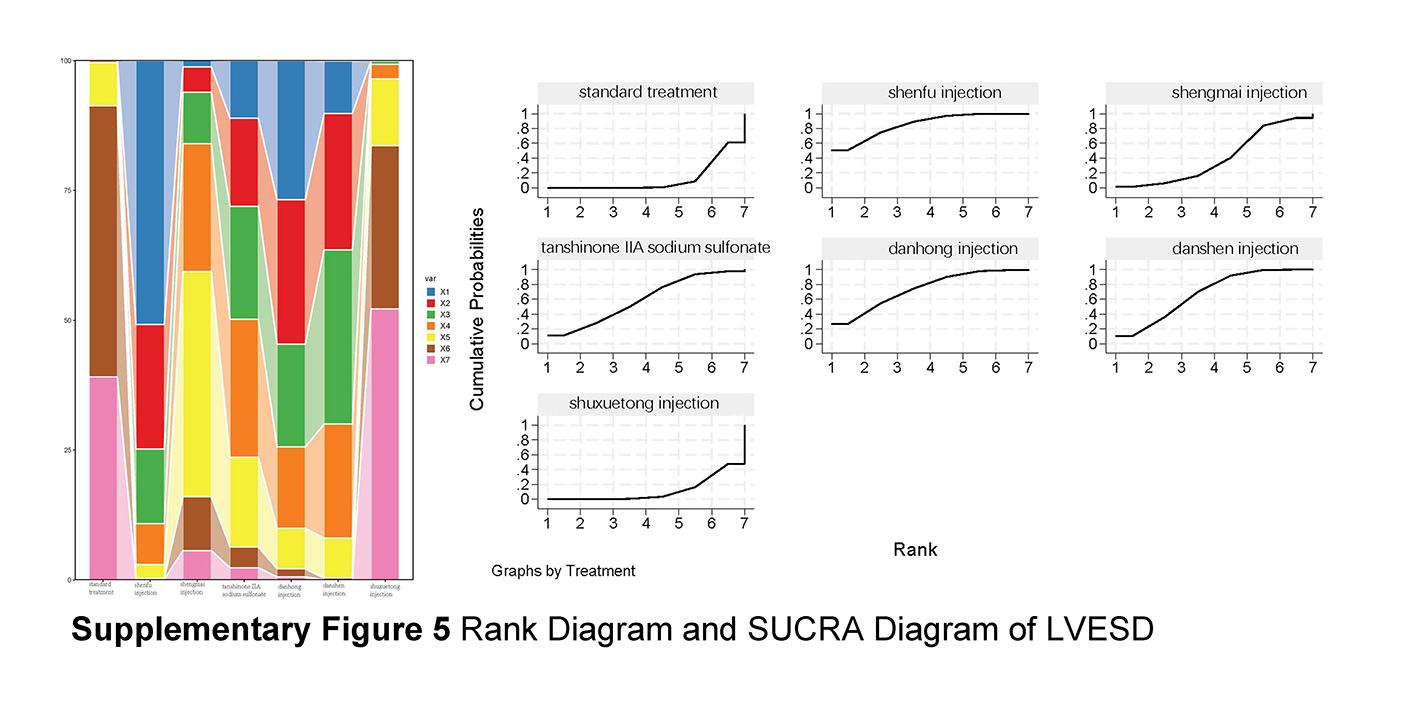

Supplement: Supplementary file 5 [file Image5.tif]

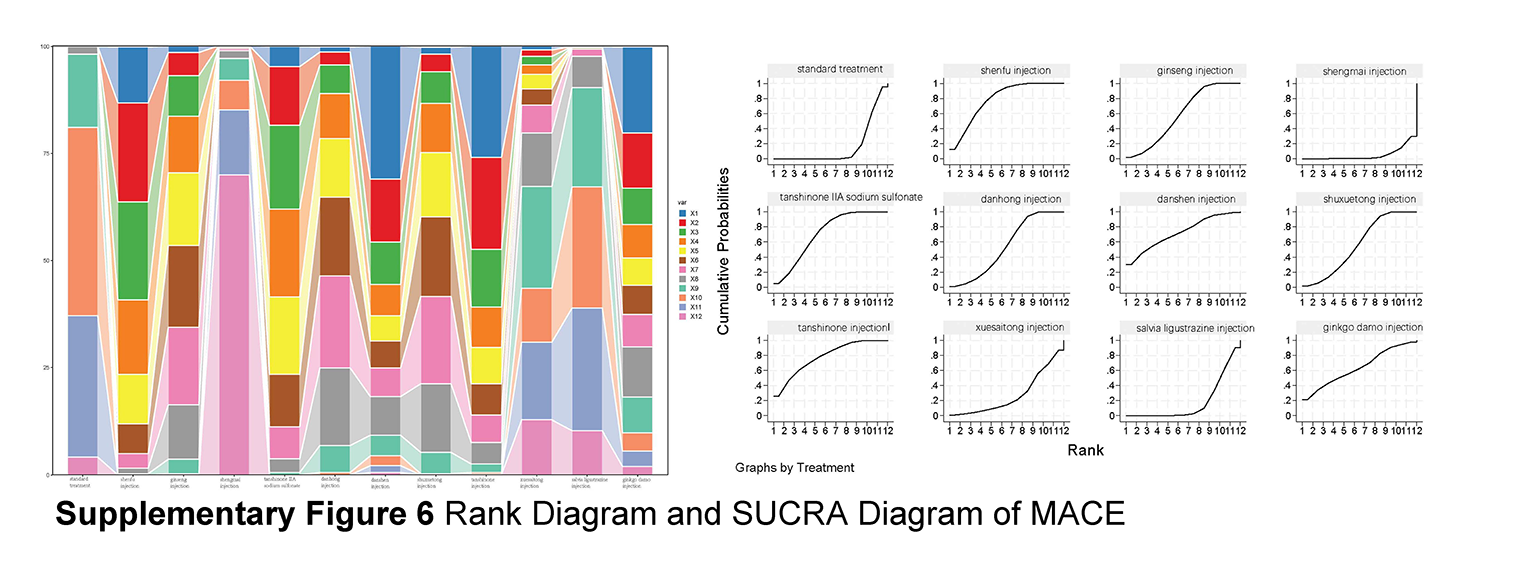

Supplement: Supplementary file 6 [file Image6.tif]
